# Bioinformatics profiling integrating a three immune-related long non-coding RNA signature as a prognostic model for clear cell renal cell carcinoma

**DOI:** 10.1186/s12935-020-01242-7

**Published:** 2020-05-13

**Authors:** Yuanbin Jiang, Xin Gou, Zongjie Wei, Jianyu Tan, Haitao Yu, Xiang Zhou, Xinyuan Li

**Affiliations:** 1 Department of Urology, The First Affiliated Hospital of Chongqing Medical University, No. 1 Youyi Road, Yuzhong District, Chongqing, 400016 China; 2 Chongqing Key Laboratory of Molecular Oncology and Epigenetics, Chongqing, China; 3 Department of Urology, Chongqing Traditional Chinese Medicine Hospital, Chongqing, China

**Keywords:** ccRCC, Immune gene, Long non-coding RNA, Risk score, Prognosis

## Abstract

**Background:**

Renal cell carcinoma (RCC) is one of the most common aggressive malignant tumors in urogenital system, and the clear cell renal cell carcinoma (ccRCC) is the most common subtype of renal carcinoma. Immune related long non-coding RNAs (IRlncRs) plentiful in immune cells and immune microenvironment (IME) are potential in evaluating prognosis and assessing the effects of immunotherapy. A completed and meaningful IRlncRs analysis based on abundant ccRCC gene samples from The Cancer Genome Atlas (TCGA) will provide insight in this field.

**Methods:**

Based on the TCGA dataset, we integrated the expression profiles of IRlncRs and overall survival (OS) in the 611 ccRCC patients. The immune score of each sample was calculated based on the expression level of immune-related genes and used to identify the most meaningful IRlncRs. Survival-related IRlncRs (sIRlncRs) was estimated by calculating the algorithm of difference and COX regression analysis in ccRCC patients. Based on the median immune-related risk score (IRRS) developed from the screened sIRlncRs, the high-risk and low-risk components were distinguished. Functional annotation was detected by gene set enrichment analysis (GSEA) and principal component analysis (PCA), and the immune composition and purity of the tumor was evaluated by microenvironment cell population records. The expression levels of three sIRlncRs were verified in various tissues and cell lines.

**Results:**

A total of 39 IRlncRs were collected by Pearson correlation analyses among immune score and the lncRNA expression. A total of 7 sIRlncRs were significantly associated with the clinical outcomes of ccRCC patients. Three sIRlncRs (ATP1A1-AS1, IL10RB-DT and MELTF-AS1) with the most significant prognostic values were enrolled to build the IRRS model in which the OS of in the high-risk group was shorter than that in the low-risk group. The IRRS was identified as an independent prognosis factor and correlated with the OS. The high-risk group and low-risk group illustrated different distributions in PCA and different immune status in GSEA. Besides, we found the more significant expression in certain ccRCC cell lines and tumor tissues of ccRCC patients compared with the HK-2 and adjacent tissues respectively. Additionally, the expression levels of lncR-MELTF-AS1 and IL10RB-DT were remarkably enhanced along the more advanced T-stages, but the lncR-ATP1A1-AS1 showed the inverse gradient.

**Conclusion:**

Our results demonstrate some sIRlncRs with remark clinical relevance show the latent monitoring and prognosis values for ccRCC patients and may provide new insight in immunological researches and treatment strategies of ccRCC patients.

## Background

With the approximate 270,000 new cases worldwide per year, renal cell carcinoma (RCC) represents 2 to 3% of all adult malignant tumors and ascends to the most common genitourinary cancer [1]. Based on the various molecular genetic features, RCC was identified as different histopathologic classifications, of which clear cell renal cell carcinoma (ccRCC) comprised the main histopathologic subtype, accounting for 70 to 80% of RCC [2].

Differ from other urogenital malignancies, ccRCC shown limited responses to chemotherapy and radiotherapy, especially for the advanced ccRCC. This motivated a series of discoveries of replacement therapies including targeted therapy and immunotherapy. Recently, some studies revealed the critical effects of immune and stromal cells on regulating tumor biological progress and ccRCC has been demonstrated remarkable immune infiltration and other immune-related signatures [3]. Therefore, an increasing number of immunotherapy drugs including PD-1/PD-L1 blocking agents have been approved in the treatment of ccRCC and inhibiting immune checkpoint shown satisfied results [4]. However, parts of patients remain response poorly, emerged resistance or progression [5]. In addition to immunotherapy, other alternative therapies, such as vascular endothelial growth factor-tyrosine kinase inhibitors, also shown their efficiency and limitations including drug resistance [6, 7]. Therefore, series of researchers are studying the underlying mechanisms, of which tumor immune escaping was regarded as one of the most probable reasons.

Consisting of immune cells, stromal cells together with other molecules, tumor microenvironment (TME), as a crucial regulator of gene expression, participated in the oncogenesis, development and prognosis [8, 9]. Although immune-related genes (IRGs) including immune-related long non-coding RNAs (IRlncRs) in ccRCC have been explored recently, and some markers plentiful in immune cells and IME are potential in assessing and predicting the sensitivity and efficacy of immunotherapy, their practical effects on prediction of prognosis and therapeutic potential remain problematic [10, 11]. Therefore, forecast the progression and prognosis of ccRCC through several novel and sensitive biomarkers might provide the more personalized guideline and more appropriate therapeutic schedule.

As a brand new class of transcripts absent of potential coding proteins, long non-coding RNAs (lncRNAs) have been demonstrated to be critically involved in the tumorigenesis, tumor progression and tumor immune response [12]. Additionally, lncRNAs significantly affect the tumor immune process including antigen exposure and recognition, as well as immune infiltration [13]. Multiple researchers identified certain dysregulated lncRNAs participating in the occurrence and progression of various tumors. LncR-SNHG1 appears to enhance the immune escaping ability in breast cancer [14]. In the context of lung cancer, lncR-NKILA was proved to display the apoptosis-promoting roles on cytotoxic T lymphocytes so that inhibited tumor immunity [15]. Besides, lncR-DILC abundant in ccRCC was identified to suppress tumor progression by stabilizing PTEN [16].

Based on the overwhelming strength and significant roles of lncRNAs on tumor immunity, their potentials of predicting progression and prognosis are being widely investigated. LncR-ZNF180-2 was examined to express significantly in advanced RCC [17]. LncR-MCM3AP-AS1 in hepatocellular carcinoma was closely associated with prognosis [18]. Although a class of studies have identified some differentially expressed lncRNAs in various tumors, the generally predictive roles of IRlncR in ccRCC remains unclear. Discovering some promising prognostic markers and investigating the underlying molecular mechanisms are eagerly anticipated.

Therefore, we designed the study to give an insight into the clinical potency of IME and IRlncRs on prognosis estimation of ccRCC. We extracted series of IRlncRs in IME forecasting poor prognosis in patients with ccRCC, and combining their clinicopathologic characteristics, we further evaluated the connections with overall survival (OS). The results shine light on clarifying the approaches and underlying mechanisms of IRlncRs on ccRCC and establish a more personalized precision predicting model for immunotherapy.

## Methods

### Human renal cell lines

ccRCC tissues and normal adjacent tissues were collected from 50 patients admitted to the First Affiliated Hospital of Chongqing Medical University and diagnosed with ccRCC. Human normal renal cell lines HK-2 and renal cancer cell lines were purchased from the American Type Culture Collection (Manassas, Virginia, USA). Cells were cultured by DMEM and 1640 medium, which were supplemented with 10% fetal bovine serum (FBS), 100 u/ml penicillin and 100 mg/ml streptomycin (Gibco, Gaithersburg, MD, USA). Incubate cells at 37 °C in 5% CO_2_. The medium was changed every 2–3 days.

### Data download and pretreatment

A series of transcriptome RNA-sequencing data of ccRCC samples were downloaded from the TCGA data portal (https://portal.gdc.cancer.gov/), that contained data from 72 non-tumor tissues and 539 ccRCC. Clinical data about these patients was also downloaded and extracted (the OS of patients ≤ 30 days were excluded because these patients probably died of unpredictable factors such as infection and hemorrhage). Raw data was prepared to do further analyses. These data were current updated in November 11, 2019. RNA-seq results were combined into a matrix file using a merge script in the Perl language (http://www.perl.org/). Next, the Ensembl database (http://asia.ensembl.org/index.html) was used to convert the Ensembl IDs of genes into a matrix of gene symbols.

### Immune-related long non-coding RNA acquisition

The Molecular Signatures Database v4.0 (Immune system process M13664, Immune response M19817, http://www.broadinstitute.org/gsea/msigdb/index.jsp) was used to specified immune-related gene participating in the immune process. Immune related gene was used to establish the immune score of ccRCC gene by GSEA. Pearson correlation analysis was used to analyse the correlation between immune score and the expression of lncRNA in ccRCC patients. IRLNRS was identified by a standard of |r| > 0.8 and P < 0.001.

### Survival-related IRlncRs (sIRlncRs)

IRlncRs associated with clinical outcomes in ccRCC patients were identified as sIRlncRs. sIRlncRs were selected by univariate COX analysis using R software survival packages(P < 0.01). Hazard ratio (HR) was used to clarify sIRlncRs into protective and deleterious portion. These sIRlncRs were specified for subsequent research.

### Create the immune-related risk score (IRRS) model

In order to verify the reliability of the sIRlncRs, sIRlncRs has been submitted for multivariate analysis, while the integrated sIRlncRs were still used as an independent prognostic indicator to develop the IRRS model. To distinguish the heterogeneous clinical prognostic outcomes, based on the differential expression of sIRlncRs, we performed a IRRS model to divide ccRCC patients into high-risk group and low-risk group. IRRS model was based on expression data multiplied by Cox regression coefficients. The formula was as followed, [Expression level of ATP1A1-AS1 * (− 0.96977)] + [Expression level of IL10RB-DT * (0.723859)] + [Expression level of MELTF-AS1* (0.035026)]. Patients were divided into low-risk groups and high-risk groups according to the median IRRS. The value of IRRS model was employed to evaluate various subtypes of ccRCC patients.

### Bioinformatics analysis

ROC curves were used to assess the sensitivity and specificity of the prognosis on the basis of IRRS. Principal component analysis (PCA) was displayed to demonstrate the expression of ccRCC samples and gene set enrichment analysis(GSEA) was used to detect the different functional phenotype between the low‐risk group and high‐risk group.

### Real-time quantitative PCR

Triazole (Invitrogen) was used to extract total RNA from tissues and cell lines under various experimental conditions according to the manufacturer’s instructions. cDNA Synthesis Kit (Osaka, Japan of TaKaRa) combining with RNA (1 μg) was utilized to reverse transcribed cDNA. The quantitative polymerase chain reaction (qPCR) was performed on an ABI 7500 real-time PCR system (Applied Biosystems) using SYBR-Green method (TaKaRa). Relative expression level of lncRNAs normalized to β-actin was calculated by the 2^−ΔCt^ method. The primer sequences are shown in Table 1. Three assays per cDNA sample.

**Table 1:** The primer sequences of IL10RB-DT, MELTF-AS1 and ATP1A1-AS1

IL10RB-DT	F primer (5′-3′)	TGCCCTACAACACCAACCCAG
	R primer (5′-3′)	ATCTTCCCGTCTAACCACTG
MELTF-AS1	F primer (5′-3′)	CCCCACAAACCTAAACAATTTGG
	R primer (5′-3′)	CCACAGAACGGTCATTCTAGTGA
ATP1A1-AS1	F primer (5′-3′)	GCCTCCTTGCCTGTGAGATG
	R primer (5′-3′)	CAAATGCACGATTTCACTCGG
β-actin	F primer (5′-3′)	AAACGTGCTGCTGACCGAG
	R primer (5′-3′)	TAGCACAGCCTGGATAGCAAC

F primer: forward primer; R primer: reverse primer

### Statistical analysis

Univariate Cox regression analysis and Pearson correlation analysis were used to identify the target IRlncRs. Kaplan–Meier curve was used to evaluate the OS between high‐risk group and low‐risk group. Univariate and multivariate Cox regression analysis were displayed to verify the independent prognostic factors for ccRCC patients. All statistical analysis was conducted using SPSS21.0 software (SPSS Inc, Chicago, IL) and GraphPad Prism5 (GraphPad Software Inc, La Jolla, CA). Varieties in clinical parameters were determined using independent t-tests. *P *< 0.05 was considered significantly statistical difference.

## Results

### Acquisition of IRlncRs

Transcriptome data and clinical data were fetched from TCGA database, and then transcriptome data was processed to convert the data ensembl ID into gene names. Following that, transcriptome data were divided into lncRNA and mRNA. From the Immune system process M13664 and Immune response M19817 of Molecular Signatures Database, we identified 331 ccRCC IRGs, of which 39 lncRNAs were validated to be the IRlncRs by correlation analysis.

### The correlation of IRlncRs and prognosis

**Fig. 1:**
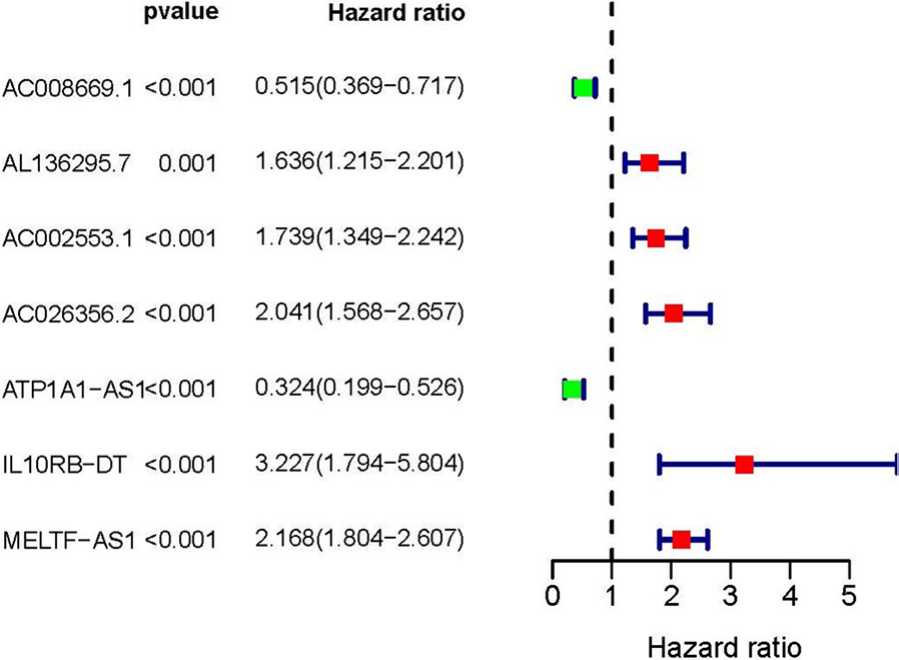
Survival-related values of sIRlncRs. Forest plot of hazard ratios showing the survival-related values of sIRlncRs (AC008669.1, AL136295.7, AC002553.1, AC026356.2, ATP1A1-AS1, IL10RB-DT and MELTF-AS1)

By COX Regression model, we then identified 7 IRlncRs which were associated with prognosis (sIRlncRs), such as AC008669.1, AL136295.7, AC002553.1, AC026356.2, ATP1A1-AS1, IL10RB-DT and MELTF-AS1. The forest map clearly illustrate the relationships between these sIRlncRs and prognosis (Fig. 1).

### Clinicopathologic characteristics of the low-risk group and the high-risk group

**Fig. 2:**
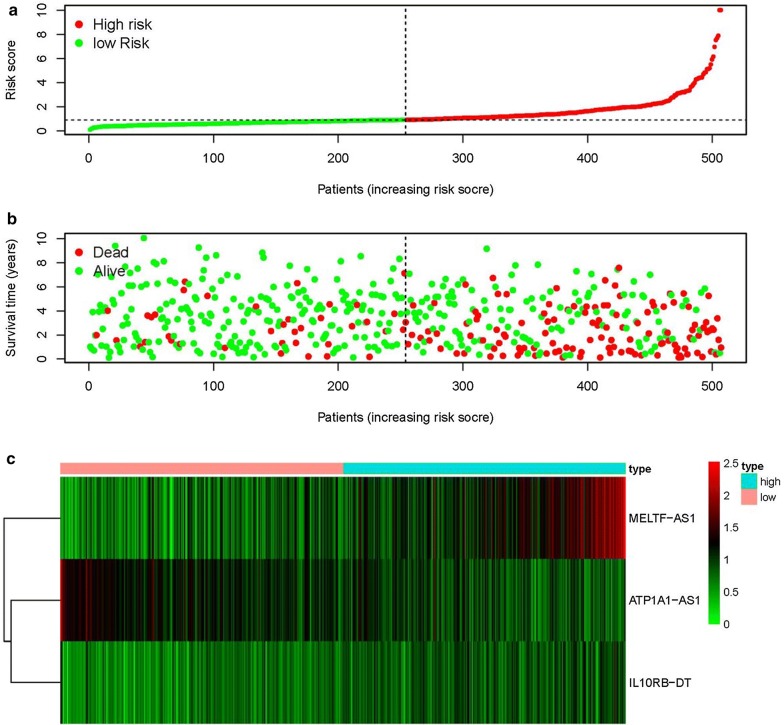
IRRS model was established according to sIRlncRs. Distribution of IRRS in high-risk group and low-risk group (**a**). Survival status between high-risk group and low-risk group (**b**). The heatmap of expression profile of contained sIRlncRs (**c**)

**Fig. 3:**
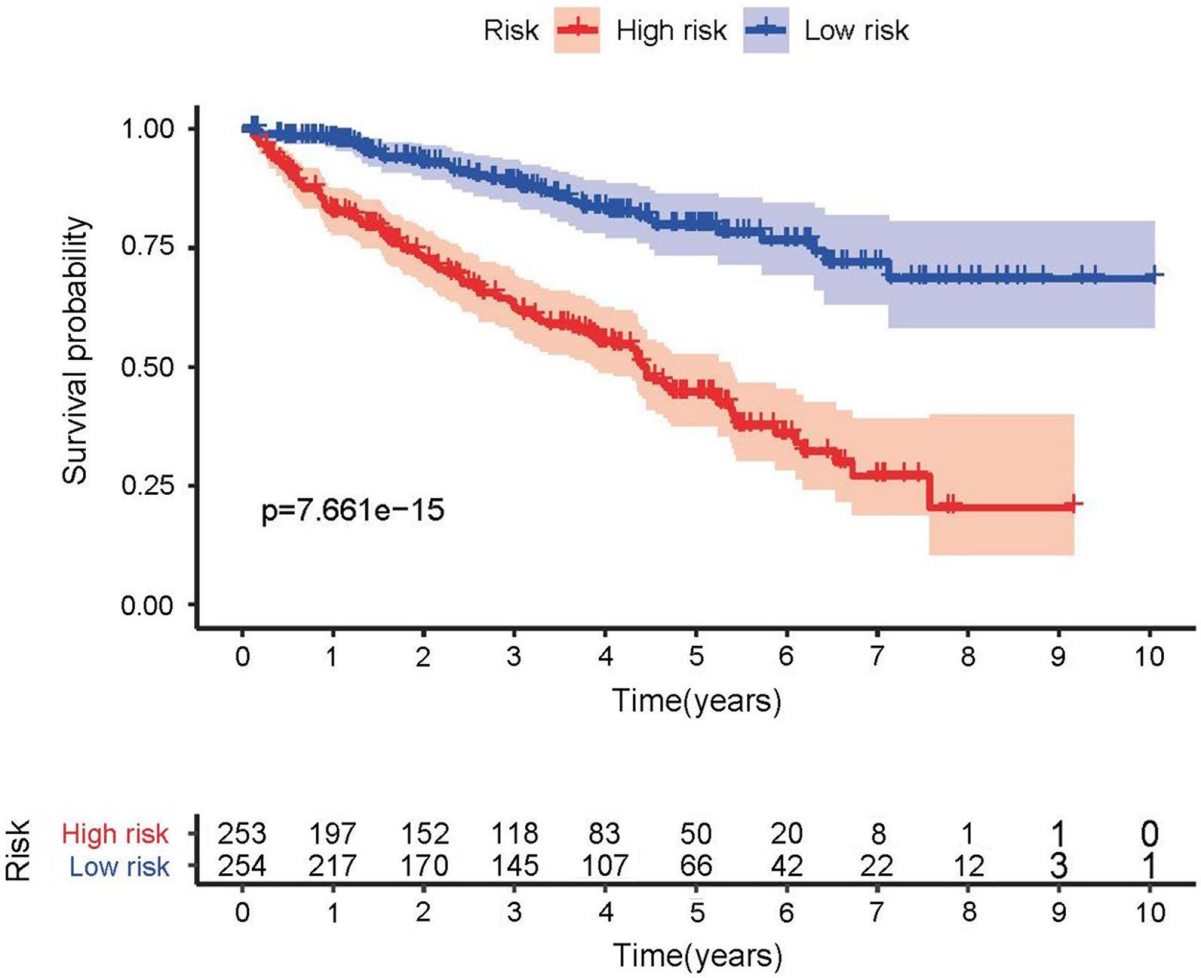
Survival curve of ccRCC patients. Kaplan‐Meier survival curve of OS among ccRCC patients from low-risk group and high-risk group. The high-risk group show the poorly prognosis

The top three sIRlncRs (ATP1A1-AS1, IL10RB-DT and MELTF-AS1) among the 7 sIRlncRs were included to establish the IRRS model, by which the ccRCC samples were divided into the high-risk group and the low-risk group (Fig. 2a). The intermediate IRRS was regarded as the critical value. The mortality rate constantly increased with the higher IRRS (Fig. 2b). And with the increase of IRRS, the expression levels of IL10RB-DT and MELTF-AS1 were elevated, while the ATP1A1-AS1 expressed decreasingly (Fig. 2c). The survival of the high-risk group was significantly shorter than that of the low-risk group (Fig. 3).

### The relationships between IRRS and clinicopathologic indicators

**Fig. 4:**
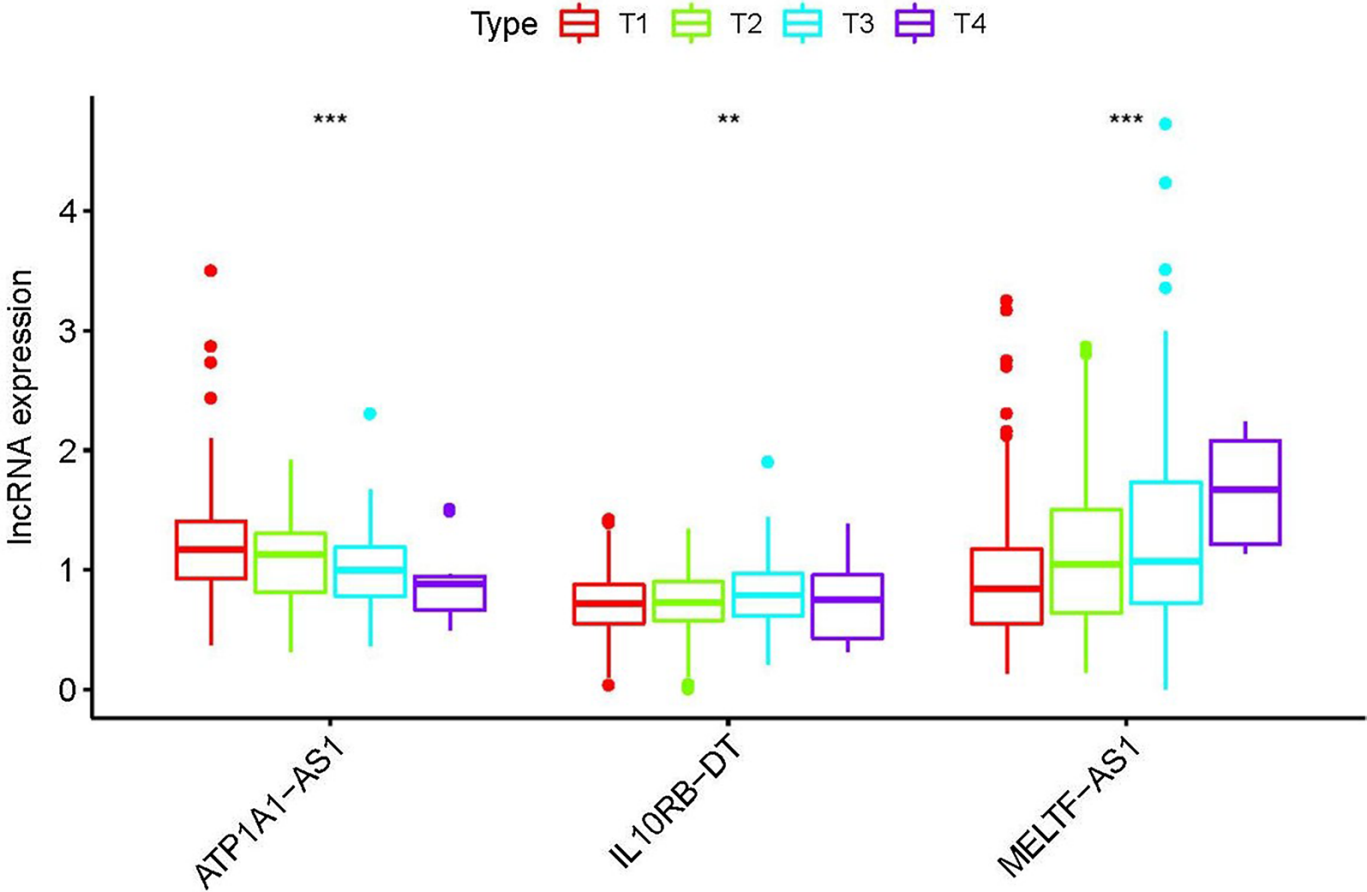
The relationships between the sIRlncRs and clinical feature. Relationships between sIRlncRs (ATP1A1-AS1, IL10RB-DT and MELTF-AS1) and T-stages. The expression of ATP1A1-AS1 decreased in the more advanced T-stages, while the expression levels of IL10RB-DT and MELTF-AS1 were enhanced

**Fig. 5:**
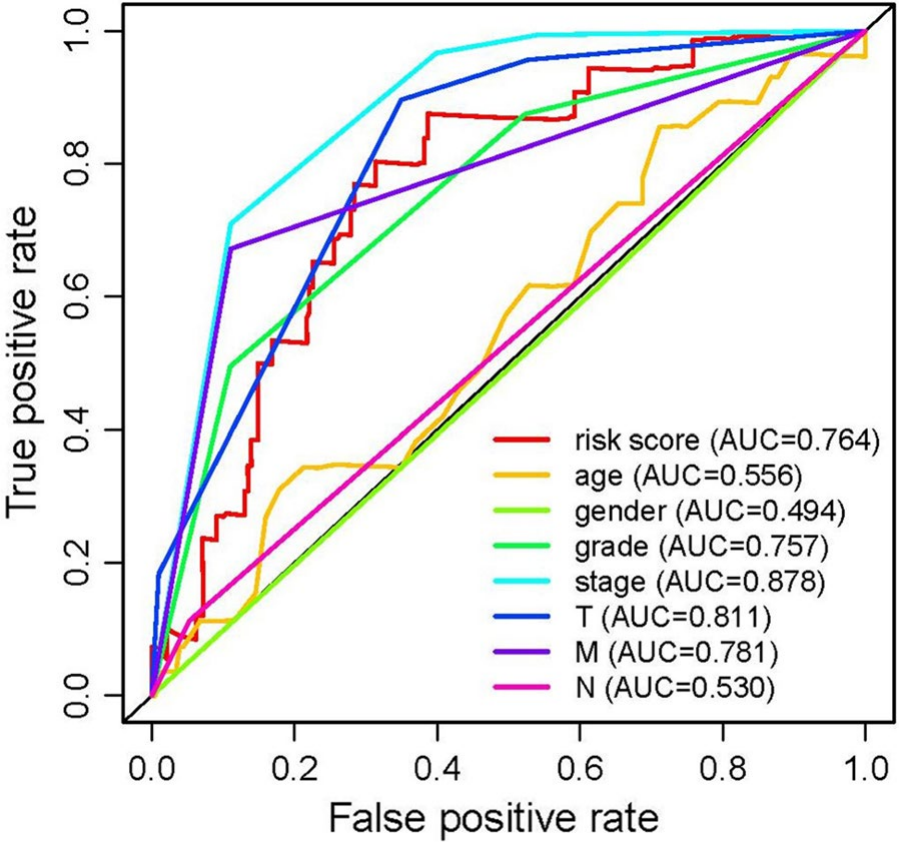
Receiver operating characteristic (ROC) curve. ROC curves indicate the prognostic value of the independent prognostic factors

To further investigate the relevance of the sIRlncRs and clinicopathological features of ccRCC, we analyzed the correlation of IRRS and the clinical and demographic characteristics, such as age, staging, grading, T-stage, N-stage and M-stage. We found the expression of ATP1A1-AS1 decreased in the more advanced T-stages, while the expression levels of IL10RB-DT and MELTF-AS1 were enhanced (Fig. 4). We then conducted the independent risk analysis, the results shown age, staging, grading, T-stage, N-stage, M-stage and IRRS were significantly correlated with OS in univariate analysis (P < 0.05). But in the multivariate analysis, only age and IRRS were remarkably correlated with OS (Table 2). The ROC curve represents the accuracy of the model. The AUC of IRRS, age, grade, staging, gender, T-stage, N-stage and M-stage are 0.764, 0.556, 0.757, 0.878, 0.494, 0.811, 0.53 and 0.781 respectively (Fig. 5). These results suggest the IRRS is an independent prognostic factor.

**Table 2:** Univariate and multivariate analysis of ccRCC

Variables	Univariate analysis				Multivariate analysis			
	HR	HR 95% low	HR 95% high	P value	HR	HR 95% low	HR 95% high	P value
Age	1.019575	1.001789	1.037678	0.030849	1.030724	1.010388	1.051469	0.002915
Gender	1.073336	0.700325	1.645024	0.745285	1.437356	0.894159	2.310543	0.134121
Grade	2.257057	1.687499	3.018849	4.10E−08	1.347571	0.956872	1.897795	0.087709
Stage	1.898909	1.566631	2.301663	6.39E−11	1.344491	0.788573	2.292313	0.276857
T-stage	1.977693	1.559362	2.508251	1.86E−08	1.052637	0.636828	1.739943	0.841430
M-stage	4.26248	2.749371	6.608325	9.12E−11	1.900099	0.835419	4.321638	0.125755
N-stage	3.035249	1.568573	5.873326	0.000978	1.416899	0.678807	2.957545	0.353347
Risk score	1.286247	1.185801	1.395202	1.29E−09	1.151379	1.045099	1.268468	0.004335

HR: hazard ratio

### Analysis of the immune status of the low and high-risk population

**Fig. 6:**
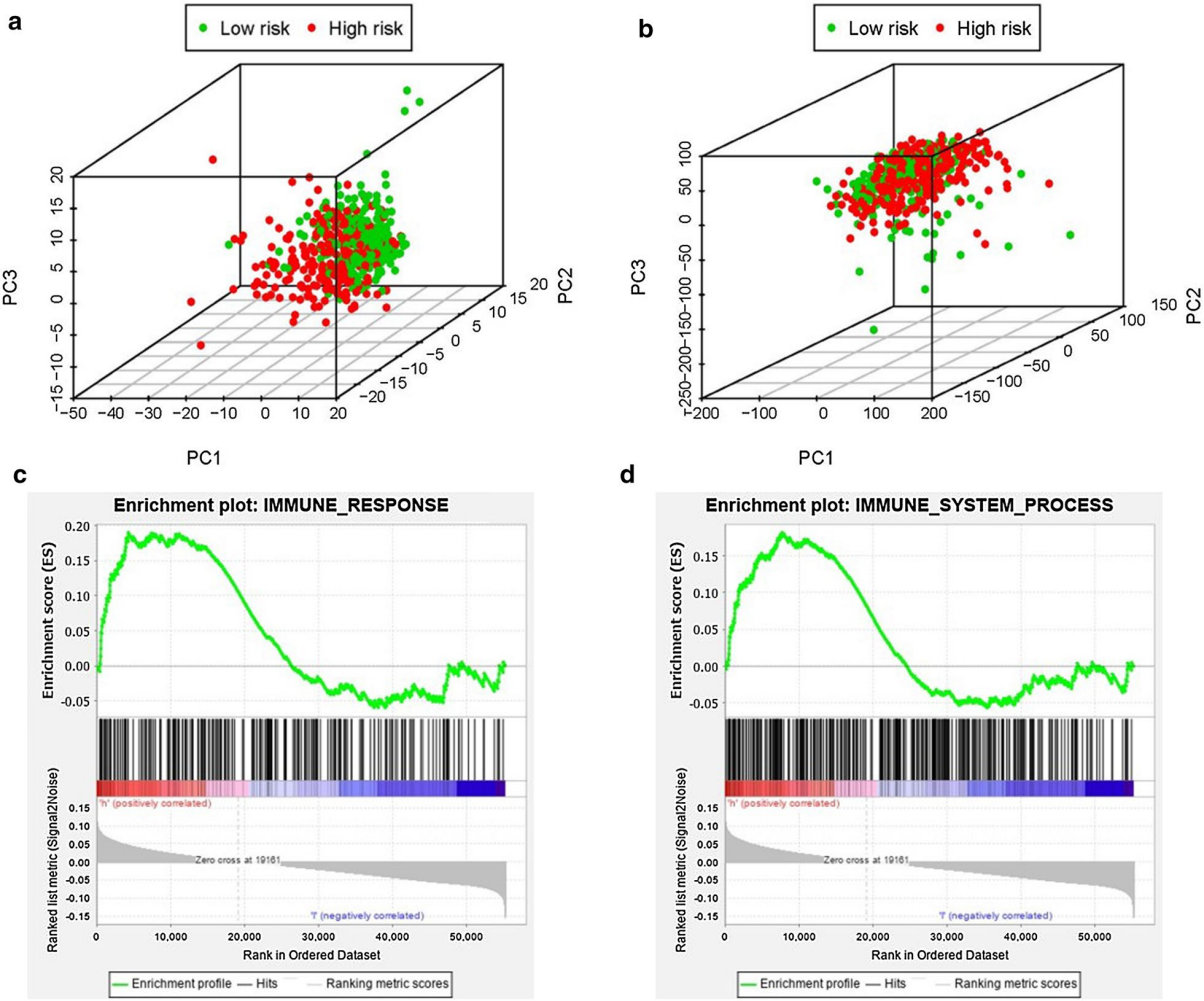
Principal components analysis (PCA) and gene set enrichment analysis (GSEA). The high‐risk group and low‐risk group demonstrated different immune status. PCA among high‐risk group and low‐risk group based on the immune‐related gene sets (**a**). PCA among high‐risk group and low‐risk group based on the whole protein‐coding gene sets (**b**). GSEA implied remarkable enrichment of immune‐related phenotype in the high‐risk group (**c** and **d**)

Based on the immune gene sets and the genome-wide expression sets, we employed the principal component analysis (PCA) to detect the different distribution patterns between the low-risk group and the high-risk group. In the IRGs set, the low-risk group and the high-risk group were separated into two parts of which the low-risk group having the lower immune scores than the high-risk group (Fig. 6a). While we didn’t detect the significant separation of the IRRS on the basis of the genome-wide expression profiles (Fig. 6b). The results of GSEA further proved the functional annotation, with the more immune-related responses and processes in the high-risk group (Fig. 6c and d).

### LncR-IL10RB-DT and MELTF-AS1 were overexpression in ccRCC patients especially with advanced T-stages

**Fig. 7:**
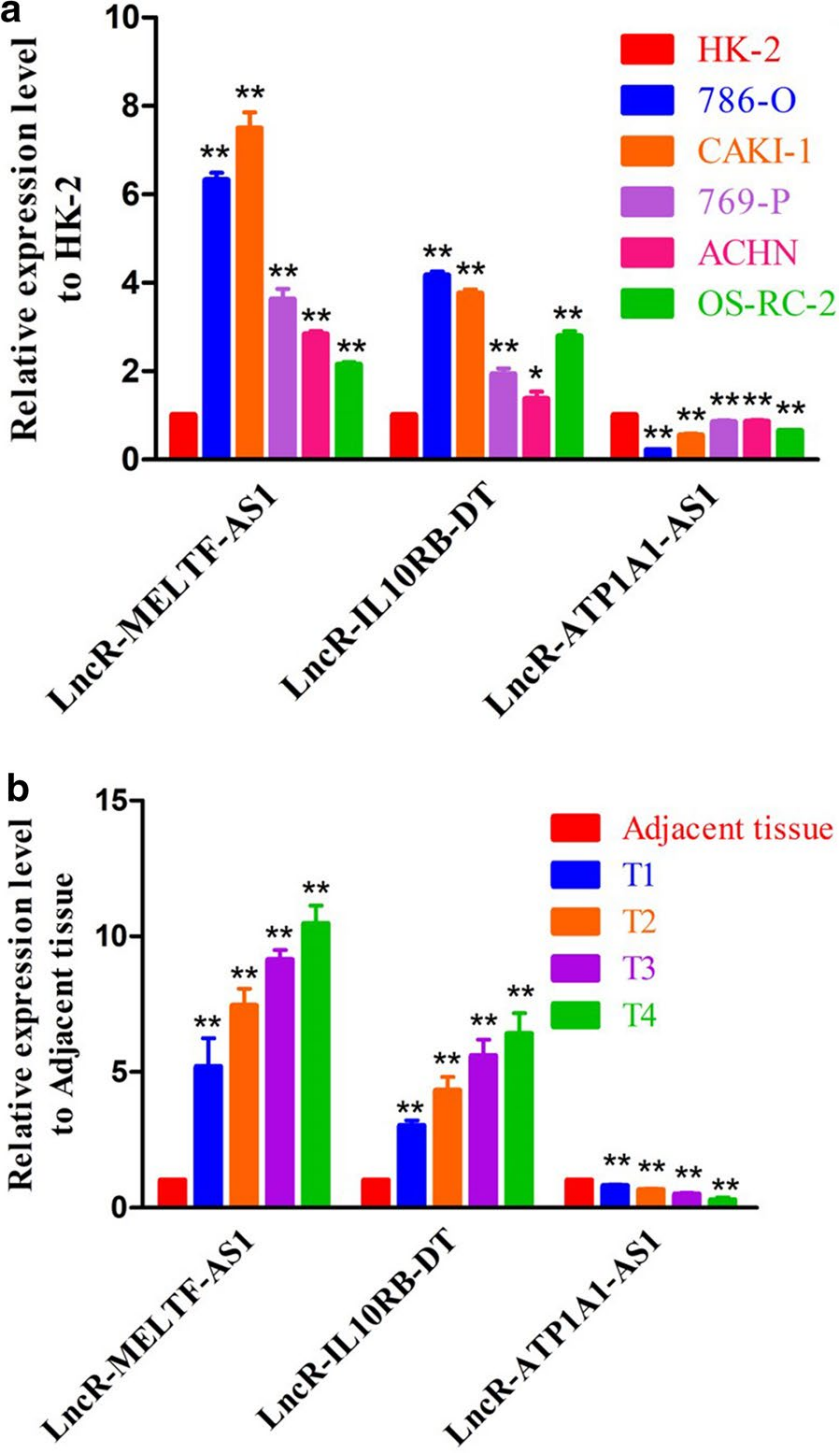
The expression levels of three sIRlncRs and their correlations with T-stages. The results of RT-qPCR of the three sIRlncRs’ expression levels in RCC cell lines (**a**), renal tubular epithelial cell (**a**), CCRCC tissues (**b**) and adjacent tissues (**b**).The expression levels of lncR-MELTF-AS1 and lncR-IL10RB-DT were higher in RCC cell lines compared to renal tubular epithelial cell, but the expression level of lncR-ATP1A1-AS1 was lower than HK-2 (**a**). ** represents the significant difference compared with HK-2 (*P *< 0.01), * represents the significant difference compared with HK-2 (*P *< 0.05). The increasing expression levels of lncR-MELTF-AS1 and lncR-IL10RB-DT were detected along with the more advanced T-stages, but the expression level of lncR-ATP1A1-AS1 decreased in the advanced T-stages than that in the earlier T-stages (**b**). ** represents the significant difference compared with adjacent tissue (*P *< 0.01), * represents the significant difference compared with adjacent tissue (*P *< 0.05)

To further verify the relationship between sIRlncRs and the clinicopathologic features, as well as discover the role of sIRlncRs on indicating clinical prognosis, we detect the expression levels of lncR-MELTF-AS1, IL10RB-DT and ATP1A1-AS1 in carcinoma and adjacent tissues of ccRCC patients with different T-stages, and among the renal tubular epithelial cell and various RCC cell lines. As illustrated in Fig. 7a, compared with HK-2, the higher expression of lncR-MELTF-AS1 and IL10RB-DT were detected in RCC cell lines including 786-O, CAKI-1, 769-P, ACHN and OS-RC-2. Consistently, carcinoma tissues showed the much higher expression levels of lncR-MELTF-AS1 and IL10RB-DT than that of adjacent tissues, besides, the expression levels were more significant in T3 and 4 stages compared with that in earlier T-stages (Fig. 7b). However, the lncR-ATP1A1-AS1 illustrated the inverse gradient (Fig. 7b).

## Discussion

The increasing cognitions about the importance of immunization activities in tumorigenesis, progression and prognosis offer more inspirations in explorations about tumor immunology, such as immunotherapy and immune-related predicted markers. Although innovated and multimodal therapeutic strategies including targeted therapy and immunotherapy have provided more novel options and prolonged survival of plenty of patients with ccRCC, the curative effects of another part of patients remain unsatisfactory [2, 6, 19]. Multiplied studies revealed the individual variation at the genetic level should be responsible for the phenomenon [20, 21]. Therefore, more explorations and understanding of ccRCC characterized by variant molecular heterogeneous, and further identifying more sensitive and effective immune prognostic indicators are above rubies.

The larger numbers of researches highlighting the vital importance of immune cells infiltration motivate us to investigate the potential of IRGs in distinguishing expression in different clinicopathologic status [22, 23]. Although certain immune-related miRNAs, mRNAs and protein indicators have been identified to forecast treatment outcomes of ccRCC and some risk models based on differentially expressed IRGs have been established to evaluate survival, increasing numbers of researches are constantly reporting that without the mission encoding proteins, lncRNAs obtain more specificity on indicating tumor actual condition than other types of markers [24]. Besides, as the main regulating factors, IRlncRs have been detected to participate in multiplied Immunization responses and activities [25]. Given the inherent strengths of lncRNAs on cancer biological processes and the remarkable immune correlation of IRlncRs, exploring their values on predicting prognosis in ccRCC patients is eagerly anticipated.

In the present study, 611 patients with ccRCC were included in a genome-wide analysis for lncRNAs, combining with 311 IRGs screened in Molecular Signatures Database v4.0 (Immune system process M13664, Immune response M19817), 39 IRlncRs were identified eventually. We further detected the relation between the prognosis of ccRCC patients and the expression levels of the 39 IRlncRs, of which 7 sIRlncRs indicated the significant correlation with OS. Utilizing multivariate Cox and risk scoring model, we further identified 3 IRlncRs to establish a risk evaluating model which was available to distinguish ccRCC patients into the high-risk group and low-risk group with obviously differences of OS. Due to the molecular heterogeneity, the accuracy and sensitivity of the present clinical risk factors in predicting the survival of ccRCC patients remain unsatisfying, we further validated the predicting value of the three sIRlncRs by multivariate analysis. We found the three sIRlncRs were independent of traditional risk factors and molecular characteristics. We also used the principal component analysis (PCA) method to study the different distribution patterns between the low-risk group and the high-risk group based on the genome-wide IRlncRs and IRGs expression set. According to the IRGs set, the low-risk group and the high-risk group tended to be divided into two parts, with the low-risk group having lower immune scores than the high-risk group. When PCA was performed on the basis of genome-wide expression sIRlncRs and IRGs profiles, the immune statues of these groups shown no significant separation. GSEA was employed to further verify the functional annotation, and we found the more abundant immune-related responses and processes in the high-risk group. Therefore, immune-related scores are bound up with the immune status of ccRCC, with higher scores indicating the poor prognosis. These findings suggest that the risk evaluating scores based on the three sIRlncRs can contribute to identify the high-risk patients from patients with the same clinical or molecular characteristics, thereby realize individualized and appropriate therapeutic strategy.

More recently, Khadirnaikar [26] and Wang [27] et al. proposed the prognostic model based on the lncR-SNHG8 and UCA1 in anaplastic gliomas and ccRCC. Chunmi et al. highlighted the predicted value of nine IRlncRs (AL138966.2, AL133520.1, AC142472.1, AC127024.5, AC116913.1, AC083880.1, AC124016.1, AC008443.5, and AC092171.5) in pancreatic cancer [28]. Meng et al. developed a risk evaluating model based on six lncRNAs (AC005013.5, UBE2R2-AS1, ENTPD1-AS1, RP11-89C21.2, AC073115.6, and XLOC_004803) to assess the prognosis of glioblastoma patients [29]. Although the characters and significance of certain IRlncRs on tumor development, progression and immune response have been established in ccRCC and other cancers, the genome-wide and completed analysis to further explore more sensitive and valued IRlncRs, especially in predicting prognosis remain sparse. In our study, a considerable number of patients with ccRCC were enrolled to improve the persuasion of clinical evidences. Besides, to further verify the relevance of sIRlncRs with tumor clinicopathologic characteristics and their prognostic values, we detected the expression levels of lncR-MELTF-AS1, IL10RB-DT and ATP1A1-AS1 in the carcinoma and adjacent tissues of ccRCC patients with various T-stages. We found the more significant expression in certain ccRCC cell lines and tumor tissues of ccRCC patients compared with the HK-2 and adjacent tissues respectively. Additionally, the expression levels of lncR-MELTF-AS1 and IL10RB-DT were remarkably enhanced along the more advanced T-stages, but the lncR-ATP1A1-AS1 showed the inverse gradient. The verification results suggest the specific sIRlncRs illustrating significant differences in variable risk factors probably indicate the progression and prognosis of ccRCC, and they are ergastic to be the personalized molecular biomarkers to assess the infiltration of immunocyte and predict treatment outcomes.

Although we validated the effects of some sIRlncRs on predicting prognosis and further verified the expression level of lncR-MELTF-AS1, IL10RB-DT and ATP1A1-AS1 in certain ccRCC cell lines and tumor tissues, there are still some limitations in the study. Firstly, in the evaluating model, the assessments of proteomics, metabolomics and immunogenomics shouldn’t be ignored to complete the more comprehensive analysis. Then, the clinical application values of these sIRlncRs remain undefined. Thirdly, in addition to lncR-MELTF-AS1, IL10RB-DT and ATP1A1-AS1, other sIRlncRs enrolled in the IRRS model also need to be detected.

## Conclusion

In the present manuscript, we comprehensively analyzed and verified the roles of the IRlncRs on indicating clinical outcomes of ccRCC. Certain sIRlncRs with remark clinical relevance were identified to be valuable for the latent monitoring and prognosis values for ccRCC patients. Our results develop a reliable and referable risk evaluating model to assess prognosis of ccRCC and provide the novel insight into immunological researches and treatment strategies.

## Data Availability

Authors can provide all of datasets analyzed during the study on reasonable request.

## Abbreviations

ccRCC: Clear cell renal cell carcinoma
FBS: Fetal bovine serum
GSEA: Gene set enrichment analysis
IME: Immune microenvironment
IRGs: Immune-related genes
IRlncRs: Immune related long non-coding RNAs
IRRS: Immune-related risk score
lncRNAs: Long non-coding RNAs
OS: Overall survival
PCA: Principal component analysis
RCC: Renal cell carcinoma
sIRlncRs: Survival-related IRlncRs
TCGA: The Cancer Genome Atlas
